# Anthropometric and Psychological Variables Associated with Body Mass Index: A Sex-Stratified Evaluation of Students from a Romanian University

**DOI:** 10.3390/healthcare14213605

**Published:** 2026-10-24

**Authors:** Ioana Mihaela Tomulescu, Mihai Ion Marian, Ciprian Brisc

**Affiliations:** 1 Department of Biology, Faculty of Informatics and Sciences, University of Oradea, 410087 Oradea, Romania; 2 Doctoral School of Biomedical Sciences, University of Oradea, 410087 Oradea, Romania; brisciprian@gmail.com; 3 Department of Psychology, Faculty of Social Sciences and Humanities, University of Oradea, 410087 Oradea, Romania; 4 Bihor Clinical County Emergency Hospital, 410169 Oradea, Romania; 5 Department of Medical Disciplines, Faculty of Medicine and Pharmacy, University of Oradea, 410071 Oradea, Romania

**Keywords:** body mass index, body adiposity index, anthropometric variables, psychosocial markers, non-invasive assessment

## Abstract

**Highlights:**

**What are the main findings?**

- Somatic association patterns: Neck circumference and Body Adiposity Index (BAI) exhibit strong concurrent associations with body mass variations across both sex sub-cohorts.
- Psychosocial metric behaviors: Subjective life satisfaction and perceived social support did not show independent significant associations with multivariable body mass status.

**What are the implications of the main findings?**

- Exploratory institutional assessment: The calculated preliminary anthropometric cut-offs provide rapid, accessible, and non-invasive evaluation indicators that could be further explored in local university student wellness assessments.
- Sex-stratified evaluation protocols: The observed dimorphism emphasizes the importance of employing sex-specific descriptive frameworks to evaluate anthropometric distributions and psychosocial parameters independently.

**Abstract:**

**Background:** Evaluating non-invasive physical markers and psychological indicators offers useful insights into young adult body profiles. This cross-sectional study evaluated the potential associations and preliminary classification capacities of select anthropometric and psychological variables in relation to body mass index (BMI) status groupings. **Methods:** A cross-sectional evaluation was conducted on a cohort of 445 university students (*n*_male_ = 199, *n*_female_ = 246) recruited from the University of Oradea. Anthropometric parameters (neck circumference, body adiposity index—BAI), a putative proxy prenatal biomarker (the right-hand R-2D:4D digit ratio), and psychosocial components (life satisfaction, total social support) were evaluated. Statistical models utilized multivariable linear regressions with collinearity diagnostics and exploratory Receiver Operating Characteristic (ROC) curve analysis. **Results:** Multivariable regressions accounted for a substantial proportion of BMI variance in males (Adjusted *R*^2^ = 0.853) and female participants (Adjusted *R*^2^ = 0.908). Neck circumference and BAI emerged as the primary statistically significant positive indicators. Standalone ROC curves suggested potential classification utility for the digital marker in both male (AUC = 0.745) and female participants (AUC = 0.805). Psychosocial metrics displayed no independent significant associations with somatic classifications. **Conclusions:** In this sample, young adult BMI categories are concurrently associated with physical parameters like neck circumference and BAI. The proposed cutting thresholds represent exploratory, internally derived thresholds specific to this convenience sample, which carry a potential mathematical optimism bias and require independent external validation before any clinical or institutional application can be evaluated.

## 1. Introduction

Obesity represents one of the most severe public health challenges of the 21st century, recognized by the World Health Organization (WHO) and global healthcare consensus frameworks as a complex, progressive chronic condition rather than a simple phenotypic variation [1]. Over the past few decades, its global prevalence has escalated to pandemic proportions, characterized by a steadily increasing rate among young adults and associated with major cardiometabolic risks, developmental impairments, and a diminished quality of life [2,3]. Although Body Mass Index (BMI) remains the universal epidemiological standard for classifying weight status, it possesses well-documented biological and structural limitations. Specifically, as a broad weight-to-height scaling ratio, BMI fails to differentiate between lean muscle mass and structural adipose tissue [4], nor does it accurately reflect or capture localized regional fat distribution. Defined fundamentally as a disproportionate and excessive accumulation of body fat relative to height, age, and sex, obesity poses a direct threat to somatic health, driving the development of metabolic syndrome and insulin resistance [5,6].

Previous literature highlights that young adulthood, particularly the university years, represents a critical period during which eating behaviours are shaped by lifestyle shifts associated with the transition to higher education [7]. Consequently, modern somatic anthropometry seeks more reliable, rapid, and non-invasive alternative or complementary assessment tools that bypass the limitations of general scaling index tracking. Integrating these markers within a sex-stratified approach is crucial given the pronounced sexual dimorphism and constitutional variations in fat distribution among young adults [8,9]. While there are numerous modalities to quantify fat excess and its bodily distribution, establishing simple, low-cost, and non-invasive anthropometric and biological indicators—such as localized upper-body subcutaneous fat deposition via neck circumference and peripheral tracking metrics via the Body Adiposity Index (BAI)—is useful. The integration of these distinct physical parameters provides a complementary structural mapping, as neck circumference reflects upper-body adiposity patterns linked to metabolic risks, whereas BAI tracks peripheral adiposity without requiring body weight metrics [10,11]. Such tools enable accessible population-based evaluations across diverse populations, simplify the identification of individuals at high risk for morbidity, and help determine priorities for both individual and community-based interventions regardless of socio-demographic contexts. Despite racial, ethnic, and environmental variations in the risks associated with specific adiposity distributions, a global consensus on obesity classification remains vital, currently relying almost exclusively on BMI and waist circumference [12,13,14].

Importantly, a comprehensive understanding of obesity cannot be confined solely to its physical or metabolic dimensions [15]. To address this, the present study utilizes a comprehensive, three-dimensional approach that integrates current somatic phenotypes, prenatal developmental proxies, and psychological health parameters. Young adults frequently navigate intense social pressures regarding body image, which can trigger psychosocial vulnerabilities. Nonetheless, the current literature yields conflicting results regarding the degree to which obesity directly co-occurs with or impairs overall well-being [16,17,18]. Within this framework, the first dimension captures immediate physical adiposity via neck circumference and BAI; the second dimension explores early developmental pathways using the right-hand R-2D:4D digit ratio as a putative or proposed proxy hypothesized to reflect prenatal hormone exposure; and the third dimension evaluates current subjective well-being through life satisfaction and perceived social support. Our rationale is that combining these distinct layers allows for an exploratory evaluation of how young adult physical traits and well-being co-vary without implying diagnostic causality. Finding precise, non-invasive alternative markers is therefore beneficial; they are highly cost-effective, require no sophisticated laboratory equipment, and minimize the psychological burden and anxiety often associated with more invasive medical evaluations [3]. Identifying stable, low-cost baseline indicators that align concurrently with BMI variations can guide subsequent long-term research trajectories and optimize standard evaluation protocols in public health initiatives and community-based assessment programs.

The aim of this cross-sectional study is to evaluate the concurrent associations among anthropometric and psychological variables within a university cohort. First, this framework analyzes the relationships between these individual indicators using linear models. Second, the analysis evaluates their exploratory classification capacities regarding body mass status within the studied cohort.

Research Objectives:

**O1.**  *To evaluate the degree of concurrent bivariate and multivariable association between continuous macro-anthropometric markers (neck circumference, BAI) and continuous BMI scores across both sex sub-cohorts.*

**O2.**  *To assess the independent, individual classification capability of the hypothesized developmental biological marker (the right-hand R-2D:4D digital ratio) relative to obesity status thresholds.*

**O3.**  *To evaluate anthropometric variables and psychological elements simultaneously within multivariable models to isolate unique variance contributions.*

**O4.**  *To calculate the preliminary classification threshold values (cut-offs) and matching Youden indices (J) for identifying obesity status based on biological sex stratification within the evaluated cohort.*

Research Hypotheses:

**H1.**  *Anthropometric indicators (neck circumference and BAI) are hypothesized to maintain significant concurrent associations with body mass variations across both sex sub-cohorts.*

**H2.**  *The right-hand R-2D:4D digital ratio is hypothesized to display exploratory classification capacities relative to obesity status thresholds, reflecting its link to early developmental parameters.*

**H3.**  *Psychological metrics (life satisfaction and perceived social support) are hypothesized to exhibit independent concurrent associations with body mass status groupings, functioning as potential co-variates within the multivariable frameworks.*

## 2. Materials and Methods

### 2.1. Study Design, Setting and Participants

This investigation utilized a cross-sectional, descriptive design to evaluate the concurrent association networks and standalone classification tracking properties of continuous somatic, prenatal, and psychosocial markers. The evaluating phase was conducted at the University of Oradea, Romania. Data collection occurred during the same period to ensure consistent conditions for all university students. Participants were selected using a convenience sampling strategy. The sample was subsequently analyzed separately for male and female students to evaluate distinct physical and psychological elements.

### 2.2. Sample Size and Sampling Procedure

To justify sample adequacy relative to the student population of the University of Oradea (*N* ≈ 22,000), Cochran’s sample size formula for finite populations was deployed. Assuming a conservative estimated proportion of *p* = 0.50 to maximize variance, a 95% confidence level (*Z* = 1.96), and a 5% margin of error (*e* = 0.05), the minimum required sample size was calculated as 378 participants.

The active final cohort utilized in this cross-sectional study comprised 445 participants, successfully exceeding the Cochran safety threshold of 378 subjects. This surplus (+17.7%) was implemented to safeguard the dataset against missing data or partially answered psychosocial instruments, thereby maximizing the numerical representativeness of the subsequent associative analyses. Following the application of specific selection filters, the final composition of the sample focused on an age range strictly constrained between 20 and 25 years resulted in 199 male participants and 246 female participants.

The following exclusion criteria were strictly applied during the initial assessment phase to eliminate confounding variables:

- Failure to report biological age or falling outside the designated age window of 20–25 years;
- Omission of biological sex tracking in the primary identity validation sheets;
- Self-reported history of major chronic metabolic conditions and/or significant cardiovascular diseases;
- Incomplete or missing responses across the applied psychosocial scaling instruments.

### 2.3. Anthropometric Measurements

To determine the target anthropometric indices (neck circumference, R-2D:4D digit ratio, BMI and BAI), the following direct anthropometric measurements were performed: standing height (H), body weight (W), hip circumference (HC), neck circumference (NC), second digit length (2D), and fourth digit length (4D).

Height (H) was measured with an ADE wall taliometer with 1 mm precision (ADE^®^ GmbH, Hamburg, Germany) with individuals lightly dressed, without shoes, standing erect, back straight, heels together and with feet slightly spread. Weight (W) was measured with a digital device using bioelectrical impedance analysis (Omron BF-511; Omron Healthcare Co., Ltd., Kyoto, Japan).

Hip circumference (HC) was measured using a flexible, non-stretchable anthropometric tape. HC was measured in centimeters as the maximum circumference over the buttocks at the level of the widest diameter around the gluteal protuberance.

Neck circumference was measured using a standardized anthropometric protocol to maximize consistency and minimize measurement artifacts. Participants were instructed to stand erect with their head positioned in the Frankfurt horizontal plane, looking straight ahead with relaxed shoulders. A flexible, non-extensible medical tape measure was applied perpendicular to the long axis of the neck, positioned just inferior to the laryngeal prominence (the Adam’s apple). Care was taken to ensure the tape maintained close contact with the cutaneous surface without compressing the underlying subcutaneous soft tissues. The measurement values were recorded at the end of a normal, unforced expiration to eliminate respiratory variance.

The length of both the index finger (2D) and the ring finger (4D) was measured directly from the metacarpophalangeal joint (the basal crease where the finger meets the palm) to the most distal tip of the fleshy pad of the finger. The fingernail was strictly excluded from the measurement. The participant placed their right hand flat on a firm, level surface, palm facing upward, with the fingers fully extended, straight, and comfortably adducted. Measurements were performed directly using a professional YATO YT-7201 (Yato Tools, Shanghai, China) stainless steel digital electronic caliper. This instrument features a linear capacitive measuring system with a high-definition LCD display, providing a resolution of 0.01 mm and an intrinsic accuracy of ±0.02 mm for measurements under 100 mm. The caliper jaws were applied gently against the anatomical landmarks to avoid skin or subcutaneous tissue compression that could artificially distort the data. Prior to taking any measurements, a comprehensive physical inspection was conducted. The investigator verified that the participant’s fingers were completely straight and entirely free of any physical deformities, congenital anomalies, joint inflammation, severe scarring, or previous orthopedic trauma that could alter the natural soft tissue profile or bone architecture.

To safeguard the dataset against human error and potential structural noise, all continuous somatic dimensions—including neck circumference and the anatomical markers required for calculating the body mass index (BMI) and body adiposity index (BAI)—were measured three consecutive times during the assessment session. The arithmetic mean of these three independent determinations was computed and integrated into the final analytical dataset, securing maximum biometric precision for the subsequent stratified regressions and classification procedures. All measurements were performed personally by a single investigator, following strict standardized anthropometric guidelines to ensure high intra-rater reproducibility and minimize measurement artifact boundaries.

Body Mass Index (BMI) is the standard epidemiological indicator utilized to evaluate whether an adult presents a healthy weight relative to height. This index is calculated by dividing body weight in kilograms by the square of height in meters [19]. The BMI metric provides a straightforward and practical approach to classify weight status, where a high value (BMI ≥ 30 kg/m^2^) classifies obesity status—a condition often associated with multiple metabolic comorbidities and increased mortality rates. The calculation formula is as follows:

$$\mathrm{BMI}=\frac{\mathrm{Weight}\;\left(\mathrm{kg}\right)}{\mathrm{Height}{\;\left(m\right)}^{2}}$$

Bergman et al. proposed a new anthropometric measurement in 2011, known as the body adiposity index (BAI), in order to estimate body adiposity variations by dividing an individual’s hip circumference in centimeters by their height in meters raised to the power of 1.5, followed by subtracting the constant 18 [20]. BAI serves as an alternative method for estimating body fat percentage. Unlike BMI, which calculates total body weight relative to height, BAI estimates adiposity levels using only hip circumference and height, with reference values varying substantially by sex. This index is often considered more precise than BMI for individuals with highly developed muscle mass, as it is not confounded by the heavy weight of lean muscle tissue. The following formula can be used to calculate BAI:

$$\mathrm{BAI}=\frac{\mathrm{Hip}\;\mathrm{circumference}\;\left(\mathrm{cm}\right)}{\mathrm{Height}\;{\left(m\right)}^{1.5}}-18$$

### 2.4. Psychosocial Measures

Psychosocial profiles were assessed using two globally anchored self-report instruments, both previously validated and extensively utilized in Romanian student populations:

Satisfaction with Life Scale (SWLS): This instrument maps the cognitive component of subjective well-being through a 5-item matrix scored on a 7-point Likert scale. Higher cumulative scores reflect a more favorable baseline evaluation of individual life trajectories. The Satisfaction with Life Scale was originally structured by Diener and colleagues to overcome critical limitations in early psychometric tools [21]. Prior measurement designs often relied on single-item assessments [22] or focused restrictedly on geriatric cohorts [23]. Additionally, legacy indexes frequently mixed distinct well-being dimensions instead of evaluating life satisfaction exclusively [24]. Within the Romanian context, the scale underwent formal psychological validation by Marian [25]. This validation framework confirmed the optimal psychometric properties and structural stability of the tool, supporting its applicability for young adult and student populations [25,26].

Multidimensional Scale of Perceived Social Support (MSPSS): This 12-item inventory measures the perceived adequacy of social support across three distinct contextual dimensions: family, friends, and significant others. Responses are recorded on a 7-point Likert scale, with higher aggregate values representing stronger psychosocial buffer networks. The Multidimensional Scale of Perceived Social Support (MSPSS) was developed by Zimet and colleagues to evaluate how individuals experience social support networks [27]. This 12-item, user-friendly instrument identifies three distinct supportive domains: family, friends, and significant others. While initial applications of this inventory included clinical cohorts, its robust psychometric properties have since justified its widespread adaptation for healthy young adults and undergraduate student populations. For the Romanian population, the MSPSS was formally validated by Marian in 2006, utilizing a cohort that included young adults over 18 years of age [28]. A separate psychometric validation in Romania was subsequently executed by Alexe and colleagues on a sample of elite athletes. Both assessment frameworks confirmed the internal consistency and stability of the scale across different active groups in the region [29].

### 2.5. Ethical Considerations

This study strictly adhered to institutional and international research ethics guidelines. All subjects participated on a completely voluntary basis and were informed in advance regarding the scope, purposes, and design of the research.

To ensure absolute privacy, participants were explicitly instructed not to provide any identifying information, such as names or personal identification codes. The protocol emphasized that only biological sex and academic program affiliation were relevant to the target exploratory population assessment. All collected data were aggregated anonymously.

The study protocol was formally reviewed and approved by the Research Ethics Committee of the Faculty of Medicine and Pharmacy, University of Oradea (Decision No. CEFMF/1 dated 28 March 2024). Furthermore, signed written informed consent was obtained from each individual participant prior to the initiation of any evaluation or anthropometric measurement procedures.

### 2.6. Statistical Analysis

Statistical analyses were conducted using IBM SPSS Statistics (Version 26.0; IBM Corp., Armonk, NY, USA). The statistical significance threshold was set at *p* < 0.05. The descriptive distribution and symmetry of continuous variables were evaluated using skewness and kurtosis indices, ensuring all calculated values fell within the accepted conservative limits of −1.00 to +1.00 to support normality assumptions required. The internal consistency of the psychosocial scales (SWLS and MSPSS) was evaluated by computing Cronbach’s alpha (*α*). All analytical procedures were stratified by biological sex (male vs. female) to analyze distinct physical and psychological elements.

The analytical sequence was structured as follows:

Descriptive and Comparative Baseline Analysis: Continuous anthropometric, digital ratio, and psychosocial variables were summarized using means and standard deviations (*SD*), while categorical variables were expressed as frequencies and percentages. To establish a detailed baseline profile of the active sample (*N* = 445), an introductory evaluation of weight status categories (underweight, normal weight, overweight, and obese) was implemented as Table 1, providing a transparent descriptive mapping of the sample weight distribution. To evaluate structural differences between male and female sub-cohorts, independent-samples *t*-tests were executed for continuous metrics, and Pearson’s Chi-Square (*χ*^2^) tests were applied for comparing categorical weight status groupings.

Bivariate Association Networks: Pearson product-moment correlation coefficients (*r*) were computed to map the direction and strength of the concurrent bivariate associations between the somatic, digital, and psychosocial markers and the continuous BMI scores.

Multivariable Structural Modeling: Multivariable linear regression analyses (Enter method) were executed to examine the concurrent associations of these variables with continuous BMI scores. To verify regression assumptions and guarantee coefficient estimation precision, multicollinearity diagnostics were rigorously tested by computing Tolerance values and Variance Inflation Factors (VIF) for each individual independent variable alongside the Durbin–Watson statistic for residual independence. Additionally, linear regression assumptions including linearity, homoscedasticity, and normal distribution of error terms were visually and statistically verified using residual scatterplots (ZRESID vs. ZPRED), and normal P-P plots, while influential observations were monitored using Cook’s distance metrics.

Classification Performance and Boundary Optimization: To measure the standalone classification capacities of the continuous markers, Receiver Operating Characteristic (ROC) curve analyses were performed. For both multivariable regressions and ROC classification structures, the epidemiological boundary for obesity status was operationalized as a Body Mass Index (BMI) ≥ 30.0 kg/m^2^, establishing a binary division (Obese vs. Non-Obese). Optimal classification threshold values (cut-offs) were determined by maximizing the Youden Index (J = Sensitivity + Specificity − 1). Confidence intervals (95% CI) were computed for the principal Area Under the Curve (AUC) estimates to evaluate estimation precision.

Pairwise Area Under the Curve Contrasts: To determine whether the computed Areas Under the Curve (AUC) differed significantly between the markers, pairwise comparison matrices were evaluated using the asymptotic DeLong method, calculating *z*-values and two-tailed significance parameters to isolate the statistical comparisons of classification performance.

The threshold for statistical significance across all mathematical models was strictly set at a two-tailed *p* < 0.05.

## 3. Results

The final evaluated cohort comprised 445 university students, with a biological sex distribution consisting of 199 male participants (44.7%) and 246 female participants (55.3%), reflecting a minor voluntary enrollment asymmetry within the university setting.

An evaluation of weight status categories across the cohort revealed significant differences in the distribution of body mass classifications between biological sexes (*χ*^2^ = 13.508, *df* = 3, *p* = 0.004), driven primarily by a higher descriptive prevalence of overweight status among male students and a higher clustering of normal weight patterns among female students (Table 1). Both sex sub-cohorts included more than 30 participants categorized within the obese bracket, providing a descriptive baseline for sub-group assessments.

Additionally, to provide a more rigorous evaluation of adiposity and address potential underestimation gaps associated with standard BMI, the Clinica Universidad de Navarra-Body Adiposity Estimator (CUN-BAE) equation was descriptively analyzed. Based on the CUN-BAE metrics, the prevalence of obesity was substantially higher compared to standard BMI classifications, identifying 62 male participants (31.2%) and 63 female participants (25.6%) within the definite obese bracket (totaling 125 participants, 28.1% of the entire cohort). A Pearson Chi-Square test indicated that the distribution of adiposity classes derived via CUN-BAE did not differ significantly between biological sexes (*χ*^2^ = 1.706, *df* = 2, *p* = 0.426).

The internal consistency of the psychosocial instruments demonstrated acceptable reliability within the total sample, yielding a computed Cronbach’s alpha (*α*) coefficient of 0.818 for the Satisfaction with Life Scale (SWLS) and 0.894 for the Multidimensional Scale of Perceived Social Support (MSPSS).

The descriptive and distributional elements of the analyzed continuous variables across sex sub-cohorts highlighted a mean Body Mass Index (BMI) of 25.35 kg/m^2^ (*SD* = 4.69) for male students and 23.77 kg/m^2^ (*SD* = 5.02) for female students, indicating an average overweight classification for the male group and a normal weight trend for the female group (Table 2). The mean neck circumference tracked at 38.46 cm (*SD* = 2.61) for male participants and 32.98 cm (*SD* = 2.66) for female participants. For the body adiposity index (BAI), female students displayed a higher mean value (*M* = 29.60, *SD* = 5.01) compared to male students (*M* = 25.77, *SD* = 4.12). The right-hand R-2D:4D digital ratio registered a mean of 0.981 (*SD* = 0.028) in men and 1.001 (*SD* = 0.027) in women. Regarding the psychological elements, life satisfaction scores showed a mean of 24.34 (*SD* = 5.63) for male participants and 25.73 (*SD* = 5.21) for female participants. Total perceived social support scores tracked at a mean of 2.23 (*SD* = 0.28) for men and 2.32 (*SD* = 0.28) for women, with a maximum value of 3.33 recorded in the male group. All calculated Skewness and Kurtosis indices fell standardly within the range of −1.00 and +1.00, supporting a reasonable approximation of symmetric data distributions for the subsequent parametric procedures (Table 2).

An independent samples *t*-test was conducted to compare biological and psychological parameters between male and female participants (Table 3). The analysis revealed statistically significant differences across all evaluated indicators. Regarding anthropometric and digital markers, male participants exhibited a significantly larger neck circumference compared to female participants [*t*(443) = 21.811, *p* < 0.001]. Conversely, female participants displayed significantly higher mean values for the R-2D:4D digit ratio [*t*(443) = −7.711, *p* < 0.001] and BAI [*t*(442.9) = −8.840, *p* < 0.001]. In terms of psychological measures, statistically significant differences were also observed, with female participants reporting higher average scores for both life satisfaction, *t*(443) = −2.698, *p* = 0.007, and total social support, *t*(443) = −3.413, *p* = 0.001, than their male participants.

Bivariate Pearson correlation analyses were conducted separately for each sex to examine the linear relationships among the biological and psychological variables (Table 4). For male participants (*n* = 199), continuous BMI scores exhibited statistically significant, strong positive correlations with macro-anthropometric indicators, namely neck circumference and the Body Adiposity Index (Table 4). A significant but weaker positive correlation was observed between BMI and the putative prenatal marker (R-2D:4D). Conversely, male BMI scores showed no statistically significant concurrent associations with psychological elements, showing negligible coupling with both life satisfaction and perceived social support networks. Concurrently, significant positive correlations were observed between neck circumference and both the 2D:4D ratio and BAI. The R-2D:4D ratio also displayed a weak positive correlation with BAI, alongside a weaker positive correlation between the R-2D:4D ratio and BAI. Among the psychological parameters, a significant negative correlation was observed between life satisfaction and total social support.

For female participants (*n* = 246), similar but more pronounced somatic association trends were identified. Continuous BMI tracking correlated very highly with neck circumference and BAI values (Table 4). The bivariate relationship between female BMI scores and the right-hand digit ratio was also positive and highly significant. In contrast to the male subgroup, female participants exhibited a statistically significant, weak negative correlation between BMI and life satisfaction, while the association with total social support networks remained non-significant. Within the wider somatic matrix of the female subgroup, neck circumference showed significant positive associations with the R-2D:4D ratio and BAI, while the R-2D:4D ratio was moderately correlated with BAI.

Additionally, female participants exhibited a significant negative correlation between BAI and life satisfaction, alongside a significant negative association between life satisfaction and total social support was also observed within the female subgroup (Table 4).

The multiple linear regression models evaluated continuous BMI variations separately for male and female sub-cohorts (Table 5). Post-estimation regression diagnostics focused on verifying standard linear modeling assumptions to ensure coefficient stability within the sex-stratified systems. Linear residual independence was supported by optimal Durbin–Watson metrics (1.98 for males, 1.95 for females). Furthermore, visual inspections of the residual scatterplots (ZRESID vs. ZPRED) and normal probability plots (P-P plots) confirmed acceptable linearity, homoscedasticity, and a normal distribution of error terms across both frameworks, while influential outlier cases were verified as non-existent based on conservative Cook’s distance minimum-to-maximum tracking limits.

The collinearity diagnostics indicated that all independent variables in both models possessed adequate Tolerance values (above 0.55) and low Variance Inflation Factors (maximum VIF = 1.29 for male students; maximum VIF = 1.81 for female students), remaining well below the conservative threshold of 5.0. This pattern indicates the absence of any severe multicollinearity inflation among the independent variables, supporting the linear estimation precision of the computed regression coefficients within this exploratory student cohort.

For male participants (*n* = 199), the global model was highly significant, accounting for 85.3% of the variance in BMI (Adjusted *R*^2^ = 0.853, *F*(5, 193) = 230.349, *p* < 0.001). Neck circumference (*B* = 0.78, *β* = 0.43, *t* = 13.99, *p* < 0.001) and BAI (*B* = 0.73, *β* = 0.64, *t* = 20.86, *p* < 0.001) were the only variables showing a significant positive association with young adult BMI scores within this cohort. The digital ratio and psychological parameters did not show independent association patterns with the dependent variable.

For female participants (*n* = 246), the regression model also displayed a substantial fit, explaining 90.8% of the BMI variance (Adjusted *R*^2^ = 0.908, *F*(5, 240) = 483.512, *p* < 0.001). Consistent with the male sub-cohort, neck circumference (*B* = 0.675, *β* = 0.357, *t* = 14.182, *p* < 0.001) and BAI (*B* = 0.679, *β* = 0.677, *t* = 25.993, *p* < 0.001) remained the sole statistically significant positive variables. Concurrently, the R-2D:4D ratio, life satisfaction, and total social support did not reach statistical significance thresholds within this multivariable context.

The individual classification performance of the analyzed anthropometric, digital, and psychological variables highlighted specific discriminative capacities when optimized via the maximum Youden index (Table 6).

**Figure 1:**
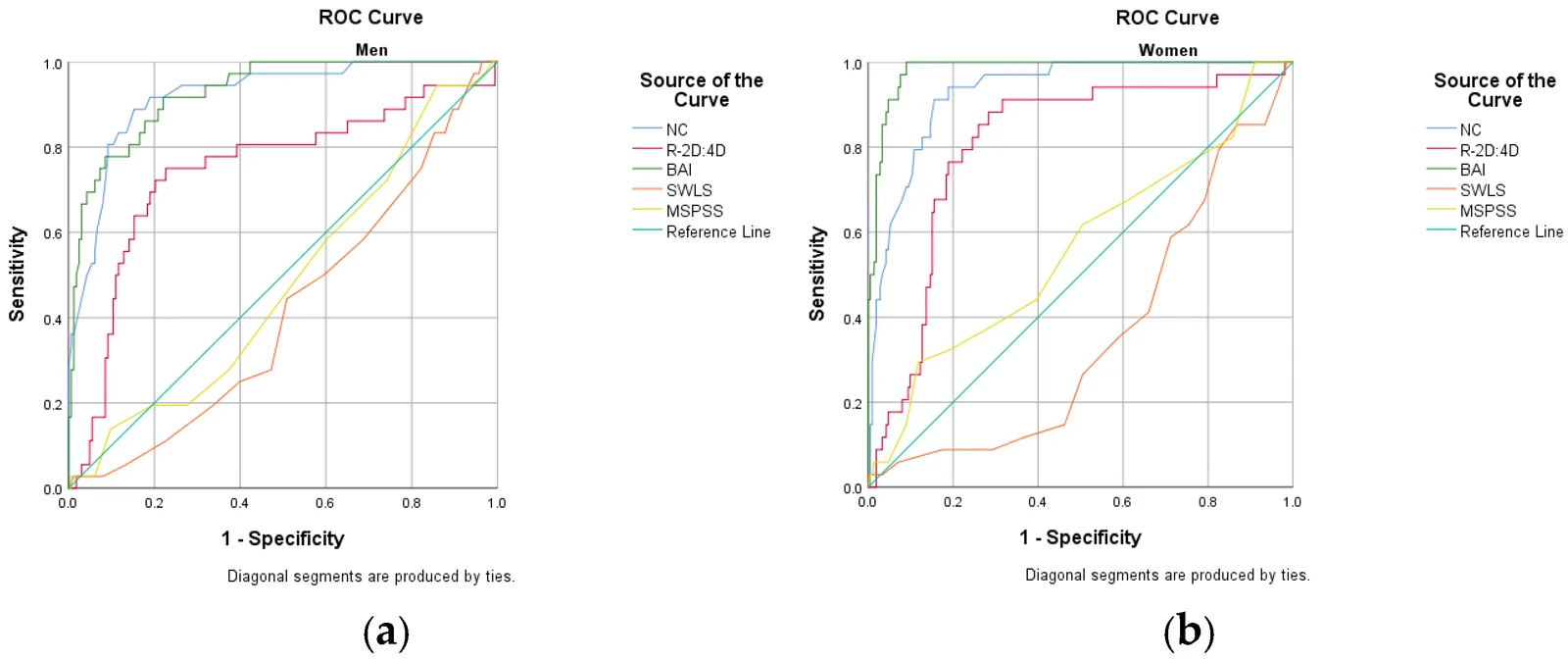
Receiver Operating Characteristic (ROC) curves evaluating the classification capacity for obesity status among university students: (**a**) male participants; (**b**) female participants. Note: NC = neck circumference; R-2D:4D = right hand 2D:4D digit ratio; BAI = body adiposity index; SWLS = Satisfaction with Life Scale; MSPSS = Multidimensional Scale of Perceived Social Support.

For male participants (*n* = 199), the non-parametric analysis established statistically significant discriminative capacities for three out of five continuous indicators relative to obesity status (Table 6, Figure 1a). The body adiposity index (BAI) displayed an area under the curve (AUC) of 0.928 (95% CI: 0.887–0.970, *p* < 0.001). The optimal cut-off point for this somatic index was established at 28.87, which maximized the classification profile with a Youden threshold of 0.692, yielding a corresponding sensitivity of 77.8% and a specificity of 91.4%. Concurrently, neck circumference (NC) showed a significant individual classification capacity, producing an AUC of 0.921 (95% CI: 0.874–0.968, *p* < 0.001). The optimal cut-off value for neck circumference was 39.90 cm, maximizing the Youden index at 0.736, providing a sensitivity of 88.9% and a specificity of 84.7%. The developmental biomarker, quantified via the R-2D:4D digital ratio, also exhibited substantial standalone discriminative utility in male participants, showing an AUC of 0.745 (95% CI: 0.645–0.845, *p* < 0.001). The mathematical optimization localized its optimal cut-off value at 0.9955 (*J* = 0.523), linking it to a sensitivity of 75.0% and a specificity of 77.3%. Conversely, psychological elements showed no independent classification capacity in the male sub-cohort (Table 6, Figure 1a). Life satisfaction tracking yielded an AUC of 0.416 (95% CI: 0.318–0.514, *p* = 0.093), with an artificial cut-off at 11.00, while total social support tracking remained non-significant with an AUC of 0.482 (95% CI: 0.381–0.583, *p* = 0.726) and an optimal cut-off value at 1.96.

Regarding continuous case classification boundaries evaluated through the optimal Youden cut-off thresholds, distinct tracking profiles emerged across both sex sub-cohorts (Table 6). Within the male sub-cohort layout, neck circumference correctly isolated 32 true positive (TP) obese cases and 138 true negative (TN) tracks, while yielding 25 false positive (FP) classifications and 4 false negatives (FN); concurrently, the male BAI matrix accurately identified 28 true positive subjects and 149 true negatives, against 14 false positive and 8 false negative tracks. Within the female sub-cohort data, the BAI framework showed high sensitivity by correctly identifying all 34 true positive obese individuals and 193 true negatives, while carrying 19 false positive tracks and 0 false negatives, whereas the female neck circumference metric isolated 23 true positive cases and 196 true negatives, yielding 16 false positive and 11 false negative classifications (Table 6).

For female participants (*n* = 246), a consistent but localized pattern emerged for the anthropometric indicators (Table 6, Figure 1b). BAI presented an individual AUC value of 0.982 (95% CI: 0.969–0.995, *p* < 0.001). The optimized female threshold for this indicator was situated at 32.73, maximizing the classification profile with a high Youden index of 0.910, which marked a sensitivity of 100.0% and a specificity of 91.0%. Additionally, the regional upper-body fat indicator, neck circumference, generated a substantial area under the curve (AUC), yielding an AUC of 0.930 (95% CI: 0.894–0.967, *p* < 0.001). The optimal cut-off value was 34.35 cm (*J* = 0.756), corresponding to a sensitivity of 91.2% and a specificity of 84.4%. The female R-2D:4D digit ratio baseline also maintained significant independent classification boundaries, tracking at an AUC of 0.805 (95% CI: 0.727–0.883, *p* < 0.001), with an optimized cut-off at 1.0107 (*J* = 0.599, sensitivity = 88.2%, specificity = 71.7%). In contrast to the male subgroup, life satisfaction scores in female participants exhibited a statistically significant, inverse discriminative pattern (Table 6 Figure 1b), yielding an AUC of 0.357 (95% CI: 0.263–0.450, *p* = 0.003), with a cut-off value at 34.50 (*J* = 0.029, sensitivity = 2.9%, specificity = 100.0%). This statistical significance aligns with the sex-specific bivariate negative correlations observed, suggesting that higher body mass indices concurrently associate with lower subjective well-being in the female sub-cohort. However, given the extremely low Youden index (*J* = 0.029) and marginal sensitivity (2.9%), this statistical significance reflects an inverse structural association rather than a viable classification tool for practical application. Total social support scores remained non-significant within the female cohort, presenting an AUC of 0.563 (95% CI: 0.452–0.673, *p* = 0.266) and an optimized cut-off at 2.63. The adjusted case-tracking diagnostics highlighted that the female BAI system achieved maximum sensitivity by correctly classifying all 34 true positive obese individuals with 19 false positives, while the localized neck circumference protocol identified 31 true positive cases alongside 33 false positive tracks (Table 6).

The asymptotic pairwise contrasts derived using the DeLong method isolated unique comparative classification patterns within each biological sex framework (Table 7). For male participants (*n* = 199), the statistical comparison between the two main anthropometric indicators, neck circumference and BAI, revealed no statistically significant difference (*z* = −0.23, *p* = 0.819, AUC difference = −0.007, 95% CI: −0.072 to 0.057), demonstrating similar classification performance between the analyzed variables. However, neck circumference displayed statistically larger area boundaries when paired with the digital marker R-2D:4D (*z* = 2.97, *p* = 0.003, AUC difference = 0.176) and exhibited a significantly larger AUC than life satisfaction (*z* = 8.27, *p* < 0.001, AUC difference = 0.505) and total social support (*z* = 7.47, *p* < 0.001, AUC difference = 0.439). The body adiposity index (BAI) showed a comparable classification capacity, generating significantly larger area coverages than the R-2D:4D ratio (*z* = −3.39, *p* = 0.001, AUC difference = −0.183), life satisfaction (*z* = 8.81, *p* < 0.001, AUC difference = 0.512), and total social support (*z* = 8.15, *p* < 0.001, AUC difference = 0.446). The digital ratio marker (R-2D:4D) maintained a statistically larger classification performance area than both life satisfaction (*z* = 4.22, *p* < 0.001, AUC difference = 0.329) and total social support (*z* = 4.12, *p* < 0.001, AUC difference = 0.263). The contrast between the two psychological variables showed no significant difference (*z* = −0.81, *p* = 0.42).

For female participants (*n* = 246), the contrast matrix exposed differences among the anthropometric variables. BAI was found to be statistically superior to the localized upper-body marker, neck circumference (*z* = −2.53, *p* = 0.012, AUC difference = −0.052, 95% CI: −0.092 to −0.012). Neck circumference remained significantly larger than the digital ratio marker R-2D:4D (*z* = 2.99, *p* = 0.003, AUC difference = 0.125), and exhibited a significantly larger AUC than life satisfaction (*z* = 11.13, *p* < 0.001, AUC difference = 0.574) and total social support (*z* = 6.55, *p* < 0.001, AUC difference = 0.368). BAI displayed a significant statistical superiority over the digital ratio baseline (*z* = −4.47, *p* < 0.001, AUC difference = −0.177), and maintained statistical superiority over both life satisfaction (*z* = 12.56, *p* < 0.001, AUC difference = 0.626) and total social support (*z* = 7.32, *p* < 0.001, AUC difference = 0.419). Within the female sub-cohort, the R-2D:4D digital indicator also exhibited a statistically larger classification capacity than life satisfaction (*z* = 7.24, *p* < 0.001, AUC difference = 0.448) and total social support (*z* = 3.42, *p* = 0.001, AUC difference = 0.242). Notably, the specific pattern of life satisfaction and total social support suggested a statistically significant, inverse classification trend within the female cohort (*z* = −2.54, *p* = 0.011, AUC difference = −0.206, 95% CI: −0.365 to −0.047).

## 4. Discussion

The observed sex distribution in our cohort, characterized by a predominance of female participants (55.3%), is an important baseline factor to consider. In cross-sectional health studies involving young academic populations, asymmetries in sample distribution by sex are frequently reported due to varying response rates. Given the well-documented sexual dimorphism in anthropometric variables and digital ratios, analyzing the cohort separately by biological sex is important. This sex-stratified approach allows for a more precise evaluation of parameters within this cohort. The baseline data align with the biological assumptions regarding sexual dimorphism. The lower R-2D:4D ratio observed in the male subgroup aligns with classical embryological literature linking this phenotype to higher prenatal testosterone exposure [30]. Similarly, the physiological variations in neck circumference between sexes correspond to standard anatomical distributions. The Skewness and Kurtosis values support an approximate characterization of the variables. Furthermore, in accordance with standard regression principles, the distribution of the residuals was evaluated using the Durbin–Watson statistics (1.98 for males and 1.95 for females), while multi-collinearity boundaries were verified via Tolerance parameters and Variance Inflation Factors (VIF). The fundamental multivariable linear regression assumptions were verified for each sex-stratified model. Visual and statistical inspections of the residual normal probability plots (P-P plots) and scatterplots confirmed linear distribution, residual independence, and homoscedasticity. Additionally, maximum Cook’s distance values (0.090 for males and 0.130 for females) demonstrated the complete absence of any influential outlier observations, collectively supporting the general applicability and coefficient stability of the linear equations.

Regarding our first research hypothesis (H1), the multivariable regression outcomes and bivariate analyses strongly support that macro-anthropometric indicators maintain significant concurrent associations with body mass variations across both sex sub-cohorts.

The comparative analysis suggests phenotypic and psychological variations between male and female participants within the study cohort. The anthropometric variations, characterized by a larger neck circumference in male participants and a higher body adiposity index (BAI) in female participants, align with standard physiological and adipose tissue distribution patterns often observed in young adult cohorts. Furthermore, the lower R-2D:4D digit ratio found in male participants corroborates well-established biological frameworks regarding intrauterine hormone exposure, where a lower ratio is associated with higher prenatal testosterone levels. Interestingly, the psychological profiles also exhibited differences, with female participants reporting higher scores in both life satisfaction and social support. Within a cross-sectional interpretation, this suggests that the phenotypic tracking of this proposed prenatal biomarker—which has been hypothesized to reflect early hormonal exposure—might manifest uniquely across the examined subgroups [31]. The high variance explained by the multivariable models (*R*^2^ = 0.853 for males and 0.908 for females) and the large AUC values derived via ROC analysis are partially influenced by mathematical construction. Because BMI, BAI, and other derivative scaling equations share height as a common denominator within their formulas, a high degree of shared variance and multicollinearity is expected by construction. This shared structural metric should be considered when evaluating the practical classification utility of these indices independently, as the very high *R*^2^ values (0.85–0.91) and AUCs largely reflect overlap by construction rather than purely independent physiological covariation.

The combined correlation matrix highlights shared biological trends alongside different psychological patterns between sexes. The positive correlation between neck circumference and the body adiposity index (BAI) in both subgroups is consistent with anthropometric literature, suggesting that upper-body subcutaneous fat deposition is associated with general body adiposity estimates. Interestingly, the positive correlation between the 2D:4D digit ratio and anthropometric metrics (neck circumference and BAI) was stronger in female participants than in male participants [32,33]. Within a cross-sectional interpretation, this suggests that the phenotypic tracking of this proposed prenatal biomarker—which has been hypothesized to reflect early hormonal exposure—might manifest uniquely across the examined subgroups.

On a psychological level, the inverse relationship between life satisfaction and social support scores in both cohorts warrants attention, suggesting a complex interplay in how young adults evaluate subjective well-being relative to social networks [34]. An interesting statistical pattern emerged regarding the negative bivariate correlation between the SWLS and MSPSS scores within this cohort. Although literature frequently reports positive associations [35], this inverse correlation might be interpreted as an indicator of specific psychosocial dynamics among young adult students at the University of Oradea. This negative co-variation could hypothetically suggest a compensatory mechanism, where individuals experiencing lower life satisfaction actively mobilize and rely more heavily on their social support networks. Alternatively, intensive social ties from family backgrounds might occasionally be perceived as overprotective, correlating concurrently with lower independent life satisfaction scores in this demographic framework. These preliminary observations imply that subjective well-being components and social support systems may relate to each other differently within this academic sample, remaining a candidate hypothesis for future longitudinal validation [36].

Additionally, a significant negative association between BAI and life satisfaction was observed in the female sub-cohort, pointing toward potential weight-related descriptive trends regarding body image and psychological well-being within this subgroup [37].

The multivariable regression outcomes suggest similar patterns between the two sub-cohorts, isolating localized upper-body subcutaneous adiposity (neck circumference) and peripheral adiposity configuration (BAI) as the main variables explaining variance in BMI. The stability of these indicators across both biological sexes indicates that anatomical features have an overriding statistical presence compared to distal developmental biomarkers (R-2D:4D) or psychological perceptions. The anthropometric variations, characterized by a larger neck circumference in male participants and a higher body adiposity index (BAI) in female participants, align with standard physiological and adipose tissue distribution patterns often observed in young adult cohorts. Specifically, the R-2D:4D ratio did not show an independent association with BMI after the inclusion of the proximal somatic variables in the multivariable equations.

The high variance explained by the multivariable models and the large AUC values derived via ROC analysis are partially influenced by mathematical construction. Because BMI, BAI, and other derivative scaling equations share height as a common denominator within their formulas, a degree of shared variance is expected. This shared structural metric should be considered when evaluating the practical classification utility of these indices independently.

Similarly, psychological dimensions (life satisfaction and total social support) showed no independent association with BMI when adjusted for biological parameters. This suggests that mental health and subjective well-being are important elements of overall health. However, body mass index variations are closely related to anthropometric and biological variables in this demographic. Methodologically, incorporating the Durbin–Watson statistics supports residual independence, while the evaluation of Tolerance parameters and Variance Inflation Factors (VIF) confirms the absence of severe multi-collinearity, collectively supporting the stability of the computed coefficients within the analyzed student sample. Additionally, the psychometric internal consistency of the instruments was supported by acceptable baseline parameters, yielding a Cronbach’s alpha (α) coefficient of 0.818 for the SWLS and 0.894 for the MSPSS within this sample.

Focusing on our second hypothesis (H2), the evaluation of individual classification capacities via standalone ROC analyses provided an exploratory overview of the parameters within this study.

The analysis of individual classification capacities provides an exploratory overview of the relationships among the evaluated parameters within this study.

Modeled independently, the right-hand R-2D:4D digital ratio shows a statistically significant capacity to classify obesity status in male participants (AUC = 0.745). This points to a preliminary association that can be hypothesized as a potential biological link between theoretical early developmental parameters and adult physical phenotypes. However, anthropometric parameters like neck circumference and BAI show much higher classification capacities (AUC > 0.920). When modeled together, in the multivariable regressions, the proximal body measurements account for the main proportion of the variance, and the independent contribution of the digital ratio is no longer significant. Furthermore, the high sensitivity and specificity metrics calculated via the optimal Youden cut-offs reinforce their classification utility. The thresholds of 39.90 cm for neck circumference and 28.87 for BAI might represent rapid, low-cost classification alternatives strictly within similar university student cohorts. In contrast, the lack of statistically significant classification capacity for the psychological constructs highlights that subjective well-being dimensions are not independently associated with somatic obesity thresholds in this demographic group. These outcomes support a highly stratified division between physical phenotypes and psychological evaluations.

This analytical pattern—where the right-hand R-2D:4D digital ratio exhibits statistically significant classification utility in the ROC analysis, yet does not retain its statistical significance within the multivariable linear models—reflects the distinct analytical purposes of these statistical methods. Evaluated individually through non-parametric ROC curves, the digital marker suggests an association with obesity status (Obese vs. Non-Obese status). However, when all variables are modeled concurrently using multivariable regressions, the macro-anatomical phenotypes (specifically neck circumference and BAI) are the main indicators explaining the variance in continuous BMI scores. Because anthropometric variables share a strong association with adult body mass parameters, the independent contribution of the digital ratio is no longer significant in the adjusted models. The pairwise comparisons evaluating the classification areas suggest differences in phenotypic performance across biological sexes. In the male sub-cohort, neck circumference and BAI scores show statistical equivalence suggesting that upper-body subcutaneous fat deposition and peripheral adiposity markers provide similar classification patterns within this lot. In contrast, differences emerged in the female sub-cohort, where the BAI index displayed a statistically larger area than neck circumference, suggesting that hip-to-height scaling parameters may classify female adipose tissue distribution with higher precision, indicating that physical measurements (neck circumference and BAI) showed higher area values than the distal developmental biomarker (R-2D:4D) in these cross-sectional structures. The pairwise comparison matrices derived using the DeLong method provided empirical evidence regarding the performance equivalence or difference of these somatic metrics. Within the male sub-cohort, the non-significant contrast between neck circumference and BAI indicates that both regional upper-body and general peripheral adiposity estimates track obesity status with comparable mathematical precision. Conversely, the significant DeLong variations identified within the female sub-cohort highlight a noticeable disparity, where the superior area boundaries achieved by the BAI index suggest that hip-to-height scaling components capture female adipose tissue configurations with higher accuracy than localized upper regional indicators. Because the study protocol was not formally pre-registered, all these extensive cross-disciplinary pairwise contrasts are interpreted strictly as exploratory parameters. Running multiple matrix tests under a standard alpha threshold carries an inherent risk of Type I error inflation (false positives). Therefore, these comparative metrics are discussed purely as parallel descriptive co-variations to ensure strict statistical caution, requiring future external population cross-validation before any clinical tracking can be inferred.

The statistically significant classification capacity of neck circumference identified across both sex subgroups in our study aligns with data reported in the literature [38,39]. Recent investigations position this regional anthropometric metric as a non-invasive indicator of adiposity that correlates closely with the body mass index profile in young adults. Specifically, Araste et al. (2025) observed that neck circumference displays a close association with cardiovascular risk factors and can support the screening of cardiometabolic syndrome [40]. Similarly, Fatnani and Narayanaswamy (2025) noted that as a low-cost and easily obtained parameter, neck circumference is significantly connected to the core components of metabolic syndrome, showing potential as a practical screening tool in resource-limited frameworks [41]. This pattern is further supported by Strathmann et al. (2025) in a cohort study from Germany, which confirmed that neck circumference is strongly associated with several cardiometabolic risk factors [42]. These cumulative observations suggest that neck circumference may serve as a useful surrogate marker for cardiometabolic risks, providing a plausible conceptual rationale for its exploration as a preliminary screening tool in healthy young populations [42].

The higher area under the curve values for BAI identified within the female sub-cohort align with studies examining sex-specific adipose tissue distribution [20,43]. This research suggests that formulas based on hip-to-height scaling can reflect variations of peripheral fat deposition in females with higher accuracy than upper regional markers. In a validation study, Lam et al. (2013) concluded that the BAI framework may underestimate body fat percentage in specific demographics and is unlikely to serve as a better overall index of adiposity than the established BMI [44]. In contrast, an investigation by Zwierzchowska et al. (2021) concluded that the BAI is complementary to BMI, recommending this tool for estimating body fat and tracking cardiometabolic risks due to its notable practical utility for general assessments [45]. Similarly, Habib et al. (2025) indicated that the BAI has a close association with body fat and could replace BMI as a classification tool in community frameworks [46]. This applicability is further supported by Ononamadu et al. (2017) who showed the potential involvement of the BAI in tracking hypertension risks [47]. Finally, Kheirouri and Alizadeh (2024) confirmed the significant classification utility of the BAI index alongside other anthropometric parameters for metabolic syndrome tracking [48].

The statistically significant discriminative capacity of the right-hand digital ratio identified in our exploratory ROC analysis—yielding an AUC of 0.745 for male participants and 0.805 for female participants—aligns with traditional biological frameworks linking prenatal hormone exposure to adult digital indicators [49]. Within this context, some investigators have also evaluated the bivariate relationship between neck circumference and the 2D:4D ratio. Specifically, Fink and colleagues evaluated a sample of 127 male and 117 female participants to analyze this association as a potential factor in coronary heart disease frameworks [50]. Consistent with the positive bivariate correlations observed in our raw data, the authors observed a significant positive correlation between the 2D:4D ratio and neck circumference in men, but not in women, after adjusting for body mass index scores. Their results indicated that a higher digital ratio aligns concurrently with a larger neck circumference baseline [50]. However, the lack of statistical significance for this biomarker within the multivariable regression models in the current study represents an important methodological outcome. Specifically, when neck circumference and BAI are included concurrently, the independent contribution of the digital ratio is no longer significant, which supports our initial hypothesis that mature adult physical dimensions are more closely and directly associated with body mass variations than early developmental markers within cross-sectional frameworks. This configuration is further discussed by recent literature investigating body weight grouping thresholds. Iljin et al. (2022) reported a significant link between adipose tissue accumulation and prenatal estrogen exposure levels, suggesting that the R-2D:4D ratio could serve as a useful exploratory factor for tracking excessive body weight risks [51]. Similarly, recent research by Yurtsever et al. (2026) indicated that bilateral 2D:4D asymmetry, particularly leftward asymmetry, may track developmental instability parameters associated with insulin resistance among women with obesity [52]. Additional studies also suggest this concurrent relationship between the R-2D:4D ratio and elevated BMI values [53].

Finally, regarding our third hypothesis (H3), multivariable frameworks revealed that psychological metrics did not maintain independent concurrent associations with body mass status groupings, leading to the rejection of H3.

The unexpected negative correlation between life satisfaction and perceived social support identified in our sample (*r* = −0.266 for male participants and *r* = −0.378 for female participants) requires cautious context, as a recent study involving 9565 Spanish adolescents indicates that BMI parameters display clear associations with subjective well-being profiles and peer interactions at school [54]. Similar to the variations observed across our sex-stratified sub-cohorts, the literature often highlights a concurrent link between lower life satisfaction scores and obesity status thresholds. A broad international investigation including participants from 34 countries across Europe, the Middle East, and Central Asia adds further nuance to quality-of-life research. This study showed that the association between BMI scores and decreased life satisfaction is partially moderated by contextual environments, with distinct patterns emerging based on biological sex [37]. Similarly, a comprehensive review by Ijeaku et al. (2026) indicated that across the lifespan, life satisfaction scores align more closely with subjective weight perception than with objective BMI classifications [55]. This association was particularly pronounced in women, aligning with the significant negative correlation between BAI and life satisfaction found exclusively among our female participants, who showed greater vulnerability to negative body weight perceptions compared to their male counterparts. Furthermore, the findings emphasize the psychological burden of perceived body image in relation to well-being parameters, irrespective of objective weight categories. Additionally, the authors highlighted the relevance of sex, geographic frameworks, and cultural factors in the interplay between life satisfaction profiles and obesity statuses [55]. This pattern is further analyzed by Kuroki [56], who reported that the statistical association between obesity and lower life satisfaction remains significant even after adjusting for socioeconomic variables and weight-related health parameters. The author also noted that effective public health policies targeting body mass assessments could support higher life satisfaction outcomes among individuals facing challenges with dietary self-control frameworks [56]. Regarding the inverse co-variation between SWLS and MSPSS observed in our student cohort, recent psychosocial literature [57,58] suggests that high levels of perceived social networks within university populations can sometimes encompass a “social support burden” or perceived pressure to conform to familial and peer expectations, which may concurrently relate to lower independent life satisfaction scores.

Presenting the explicit case classification layout provides greater methodological transparency regarding the performance of these cutting thresholds. The empirical evaluation of true positive (TP) and false positive (FP) metrics highlights that the female BAI system identified the 34 obese individuals within this sample, alongside 19 false positive tracks. Within the male sub-cohort, neck circumference isolated 32 true positive cases against 4 false negatives, while yielding 25 false positive classifications. These tracking frameworks indicate that while both somatic tools present high descriptive classification utility for this group, they carry over-classification tendencies that reflect the uncertainty of individual cut-off values in relatively small sub-cohorts.

Regarding social support networks, the current literature indicates their general relevance within weight status evaluations. For instance, in an investigation of 188 Mexican adolescents, Guevara-Valtier et al. (2021) concluded that a lack of motivation serves as a perceived barrier to optimal dietary tracking and physical activity [59]. Furthermore, they noted that social networks represent a useful tool for healthcare staff to promote community-based interventions and prevent metabolic conditions within global digital frameworks [59]. Similarly, a very recent study involving 457 young adults with metabolic dysfunction showed that weight self-stigma and depressive symptoms operate within a mediating role in the relationship between social support systems and emotional eating patterns among overweight and obese patients [60]. Recently, Smith et al. (2020) reported that individual body weight parameters and dietary behaviors co-vary with active social ties [61]. Additionally, the authors stated that specific underlying mechanisms, such as social norms, comparison, or behavioral modeling, require further cross-sectional evaluation across different demographic groups and life stages [61].

Through these combined statistical frameworks, the analysis indicates that body mass classifications and psychosocial dimensions did not show independent associations within this multivariable context. Obesity status classifications do not display linear associations with lower life satisfaction scores or diminished social support networks within this specific sample of students from the University of Oradea. These outcomes suggest that within this exploratory cohort, young adults with an elevated BMI do not uniformly report lower life satisfaction scores. In conclusion, while no independent associations between the psychosocial scales (SWLS and MSPSS) and BMI thresholds were detected in the present multivariable models, this general independence must be qualified by the descriptive bivariate trends, which highlighted a significant inverse association between adiposity and life satisfaction within the female subgroup.

### 4.1. Recommendations for Future Research

Explore low-cost university assessment tools: Future student-focused health surveys could explore the deployment of neck circumference and BAI as rapid, preliminary candidate indicators. These markers require no expensive laboratory equipment, representing accessible options for community-based student assessment programs.

Consider sex-specific frameworks: It is important for exploratory classification studies to account for biological sex dimorphism. Body composition assessments in female young adults could benefit from hip-based variables like the BAI.

Explore evaluated somatic thresholds: Future research and wellness programs could explore the specific anthropometric cut-offs established in this study. The optimal sex-specific thresholds computed for neck circumference and the body adiposity index may serve as preliminary operational boundaries for body mass stratification strictly within similar student populations.

Analyze evaluation parameters independently: It is useful for future research protocols to analyze somatic phenotypes and psychosocial dimensions independently, as subjective well-being profiles did not show independent linear associations with objective body mass indicators within this sample.

Apply multi-method statistical designs: Future cross-sectional research could combine multivariable adjustments with standalone classification models to provide a broader description of how developmental and anthropometric variables relate to body mass indices.

### 4.2. Limitations of the Study

This study presents several methodological limitations that should be acknowledged:

Cross-Sectional Research Design: Data were collected at a single point in time. Consequently, this framework cannot establish temporal sequences or long-term causal relationships between the somatic, biological, and psychosocial variables. The multivariable regression models reflect concurrent associations within this exploratory cohort rather than prospective pathways.

Specificity of the Sample Cohort and Convenience Sampling: The investigation was conducted on a specific group of young adult university students at the University of Oradea based on a convenience sample. Therefore, the direct extrapolation of the calculated classification thresholds to the general population or to other distinct age groups must be approached with caution, representing a limitation in generalizability beyond this sample. Additionally, the cohort may have encompassed a limited number of severe obesity cases within each sex subgroup, which could constrain the variance parameters of the equations, acknowledging the inherent statistical uncertainty and margin of error associated with relatively small clinical subgroup sample sizes in both multivariable and ROC estimations.

Absence of an Independent Adiposity Criterion and External Validation: The study lacked a direct, independent reference standard for body adiposity or metabolic health, such as dual-energy X-ray absorptiometry (DEXA) or laboratory evaluations, relying strictly on body mass index classifications. Moreover, the optimal cutting thresholds derived via the Youden index framework were evaluated internally within the same dataset without independent cross-validation or bootstrapping techniques, which introduces a potential optimism bias in the resulting sensitivity and specificity estimates. Furthermore, while essential linear regression assumptions were verified through visual inspection of residual probability plots and scatterplots alongside Cook’s distance monitoring, more stringent mathematical normal distribution checks for errors were not performed, which represents an analytical limitation.

Mathematical Overlap and Metric Interdependence: An important theoretical element involves the mathematical overlap between BMI and BAI, as both anthropometric indices incorporate height parameters within their equations. Because both metrics share this scaling baseline, their baseline co-variation represents a methodological characteristics that requires cautious contextual interpretation when evaluating multivariable regression variance (*R*^2^) fields.

Self-Reported Nature of Psychometric Data: The SWLS and MSPSS scales are instruments based entirely on the subjective perception of the participants. The potential influence of social desirability bias on the collected responses cannot be completely ruled out, and the exploratory design did not apply multiple comparison corrections to the standalone bivariate testing matrix.

## 5. Conclusions

Within the boundaries of this investigated cohort, physical measurements occupy the primary positions among the analyzed variables. Specifically, neck circumference and BAI display significant concurrent associations with BMI, reflecting a structural pattern that remains consistent across both male and female young adult sub-cohorts. In contrast, the proxy prenatal marker displays classification utility only in single-variable models; the right-hand R-2D:4D digital ratio is associated with obesity status when evaluated independently, but does not remain statistically significant when physical measurements are included simultaneously in multivariable regression equations. Furthermore, the evaluated psychosocial factors showed no independent associations with BMI in the multivariable models analyzed. Nevertheless, while these subjective psychological dimensions demonstrated limited independent overlap within the multivariable matrices, this general independence must be qualified by the descriptive bivariate trends, which highlighted a significant inverse association between adiposity metrics and life satisfaction scores within the female sub-cohort. Ultimately, the specific threshold values determined in this study may function strictly as potential candidate thresholds for future prospective investigations, serving exclusively as preliminary exploratory indicators that require extensive independent external validation.

## Figures and Tables

**Table 1:** Distribution of weight status categories based on BMI stratified by biological sex (*N* = 445).

Weight Status Category (BMI)	Male Participants (*n* = 199) Frequency/(%)	Female Participants (*n* = 246) Frequency/(%)	Total Cohort (*N* = 445) Frequency/(%)	Chi-Square Test (*χ*^2^)
Underweight (BMI < 18.50 kg/m^2^)	10/5.0	24/9.8	34/7.6	*χ*^2^ = 13.508 *df* = 3 *p* = 0.004
Normal weight (BMI: 18.50–24.99 kg/m^2^)	99/49.7	149/60.6	248/55.7	
Overweight (BMI: 25.00–29.99 kg/m^2^)	54/27.1	39/15.9	93/20.9	
Obese (BMI ≥ 30.00 kg/m^2^)	36/18.1	34/13.8	70/15.7	

**Table 2:** Descriptive and distributional characteristics of the study variables by sex (*N* = 445).

Sex/Variable	Statistics	BMI	NC	R-2D:4D	BAI	SWLS	MSPSS
Male participants (*n* = 199)	Mean	25.35	38.46	0.981	25.77	24.34	2.23
	*SD*	4.69	2.61	0.028	4.12	5.63	0.28
	Skewness	0.67	−0.04	−0.09	0.48	−0.62	0.66
	Kurtosis	0.28	0.21	−0.31	−0.25	0.26	0.96
	Minimum	16.73	30.20	0.909	17.28	7.00	1.42
	Maximum	42.13	46.10	1.058	36.24	35.00	3.33
Female participants (*n* = 246)	Mean	23.77	32.98	1.001	29.60	25.73	2.32
	*SD*	5.02	2.66	0.027	5.01	5.21	0.28
	Skewness	0.98	0.63	−0.11	0.91	−0.45	0.37
	Kurtosis	0.58	0.03	−0.41	0.66	−0.54	0.03
	Minimum	14.40	27.30	0.927	19.43	12.00	1.67
	Maximum	41.32	41.30	1.065	46.56	35.00	3.17

Note: NC = neck circumference; R-2D:4D = right hand 2D:4D digit ratio; BAI = body adiposity index; SWLS = Satisfaction with Life Scale; MSPSS = Multidimensional Scale of Perceived Social Support.

**Table 3:** Sex-based comparison of biological and psychological indicators across the total study sample (*N* = 445).

Biological and Psychological Parameters	*t*-Value	(*df*)	*p*-Value	Comparison Direction (Participants)
NC	21.811	443	<0.001	Male > Female *
R-2D:4D	−7.711	443	<0.001	Male < Female *
BAI	−8.840	442.9	<0.001	Male < Female *
SWLS	−2.698	443	0.007	Male < Female *
MSPSS	−3.413	443	0.001	Male < Female *

Note: NC = neck circumference; R-2D:4D = right hand 2D:4D digit ratio; BAI = body adiposity index; SWLS = Satisfaction with Life Scale; MSPSS = Multidimensional Scale of Perceived Social Support. * Indicates statistically significant differences at the *p* < 0.05 level based on the independent-samples *t*-test.

**Table 4:** Combined Pearson correlation matrix of study variables for male and female participants (*N* = 445).

Sex	Nr.	Variable	BMI	NC	R-2D:4D	BAI	SWLS	MSPSS
Male participants *n* = 199	1.	BMI	—	0.724 **	0.238 **	0.837 **	−0.072	−0.009
	2.	NC	0.724 **	—	0.214 **	0.443 **	−0.042	−0.052
	3.	R-2D:4D	0.238 **	0.214 **	—	0.188 **	−0.128	0.072
	4.	BAI	0.837 **	0.443 **	0.188 **	—	−0.083	0.057
	5.	SWLS	−0.072	−0.042	−0.128	−0.083	—	−0.266 **
	6.	MSPSS	−0.009	−0.052	0.072	0.057	−0.266 **	—
Female participants *n* = 246	1.	BMI	—	0.788 **	0.449 **	0.909 **	−0.175 **	0.086
	2.	NC	0.788 **	—	0.388 **	0.617 **	−0.086	0.048
	3.	R-2D:4D	0.449 **	0.388 **	—	0.410 **	−0.061	−0.043
	4.	BAI	0.909 **	0.617 **	0.410 **	—	−0.232 **	0.101
	5.	SWLS	−0.175 **	−0.086	−0.061	−0.232 **	—	−0.378 **
	6.	MSPSS	0.086	0.048	−0.043	0.101	−0.378 **	—

Note: NC = neck circumference; R-2D:4D = right hand 2D:4D digit ratio; BAI = body adiposity index; SWLS = Satisfaction with Life Scale; MSPSS = Multidimensional Scale of Perceived Social Support. ** Correlation is significant at the 0.01 level (2-tailed).

**Table 5:** Multiple linear regression models modeling body mass index (BMI) by sex (*N* = 445).

Sex/Independent Variables	Unstandardized Coefficient (*B*)/(*SE*)	Standardized Coefficient *β*	*t*-Value	*p*-Value	95% CI for *B*	Part/Cohen’s *f*^2^	Collinearity Statistics Tolerance/VIF
Male participants (*n* = 199)	*R* = 0.93 *R*^2^ = 0.86; *R*^2^*_Adj_* = 0.85; *F* = 230.35, *p* < 0.001; Durbin–Watson = 1.98						
(Constant)	−26.823 (4.932)	—	−5.44	<0.001	[−36.55, −17.09]		—
NC	0.78 (0.06)	0.43	13.99	<0.001	[0.67, 0.89]	0.38/1.01	0.78/1.29
R-2D:4D	4.59 (4.81)	0.03	0.96	0.341	[−4.89, 14.07]	0.03/0.01	0.93/1.08
BAI	0.73 (0.04)	0.64	20.86	<0.001	[0.66, 0.79]	0.57/2.25	0.79/1.27
SWLS	−0.003 (0.02)	−0.00	−0.14	0.886	[−0.05, 0.04]	−0.00/0.00	0.92/1.09
MSPSS	−0.44 (0.47)	−0.03	−0.94	0.349	[−1.38, 0.49]	−0.03/0.01	0.92/1.09
Female participants (*n* = 246)	*R* = 0.95 *R*^2^ = 0.91; *R*^2^*_Adj_* = 0.91; *F* = 483.51, *p* < 0.001; Durbin–Watson = 1.95						
(Constant)	−25.82 (4.07)	—	−6.34	<0.001	[−33.85, −17.79]		—
NC	0.68 (0.05)	0.36	14.18	<0.001	[0.58, 0.77]	0.28/0.84	0.59/1.69
R-2D:4D	6.42 (4.09)	0.03	1.57	0.117	[−1.63, 14.48]	0.03/0.01	0.79/1.26
BAI	0.68 (0.03)	0.68	25.99	<0.001	[0.63, 0.73]	0.50/2.82	0.55/1.81
SWLS	0.02 (0.02)	0.02	0.84	0.401	[−0.02, 0.06]	0.02/0.00	0.82/1.23
MSPSS	0.16 (0.38)	0.01	0.42	0.674	[−0.59, 0.91]	0.01/0.00	0.85/1.18

Note: NC = neck circumference; R-2D:4D = right hand 2D:4D digit ratio; BAI = body adiposity index; SWLS = Satisfaction with Life Scale; MSPSS = Multidimensional Scale of Perceived Social Support.

**Table 6:** ROC curve characteristics, optimal cut-off values, and case classification for identifying obesity status by sex.

Sex	Variable	AUC	95% CI	*p*-Value	Optimal Cut-Off	Youden (J)	Sens (%)	Spec (%)	TP (*n*)	FP (*n*)	TN (*n*)	FN (*n*)
Male (*n* = 199)	NC	0.921	[0.874, 0.968]	<0.001	39.90 cm	0.736	88.9	84.7	32	25	138	4
	BAI	0.928	[0.887, 0.970]	<0.001	28.87	0.692	77.8	91.4	28	14	149	8
	R-2D:4D	0.745	[0.645, 0.845]	<0.001	0.9955	0.523	75.0	77.3	27	37	126	9
	SWLS	0.416	[0.318, 0.514]	0.093	11.00	0.037	100.0	3.7	36	157	6	0
	MSPSS	0.482	[0.381, 0.583]	0.726	1.96	0.085	94.4	14.1	34	140	23	2
Female (*n* = 246)	NC	0.930	[0.894, 0.967]	<0.001	34.35 cm	0.756	91.2	84.4	31	33	179	3
	BAI	0.982	[0.969, 0.995]	<0.001	32.73	0.910	100.0	91.0	34	19	193	0
	R-2D:4D	0.805	[0.727, 0.883]	<0.001	1.0107	0.599	88.2	71.7	30	60	152	4
	SWLS	0.357	[0.263, 0.450]	0.003	34.50	0.029	2.9	100.0	1	0	212	33
	MSPSS	0.563	[0.452, 0.673]	0.266	2.63	0.176	29.4	88.2	10	25	187	24

Note: NC = neck circumference; R-2D:4D = right hand 2D:4D digit ratio; BAI = body adiposity index; SWLS = Satisfaction with Life Scale; MSPSS = Multidimensional Scale of Perceived Social Support; Sens = Sensitivity; Spec = Specificity; TP = True Positives; FP = False Positives; TN = True Negatives; FN = False Negatives. Proportions are based on the actual sample sizes stratified by obesity status (Men: 36 Obese/163 Non-Obese; Women: 34 Obese/212 Non-Obese).

**Table 7:** Pairwise comparison matrix of areas under the ROC curves (AUC) for identifying obesity status by sex.

Sex	Test Result Variable Pair	Matrix Statistics *z*-Value/*p*-Value	AUC Difference	Asymptotic 95% CI [Lower, Upper]
Male (*n* = 199)	NC—R-2D:4D	2.97/0.003	0.176	[0.060, 0.292]
	NC—BAI	−0.23/0.819	−0.007	[−0.072, 0.057]
	NC—SWLS	8.27/<0.001	0.505	[0.385, 0.624]
	NC—MSPSS	7.47/<0.001	0.439	[0.324, 0.554]
	R-2D:4D—BAI	−3.39/0.001	−0.183	[−0.289, −0.077]
	R-2D:4D—SWLS	4.22/<0.001	0.329	[0.176, 0.482]
	R-2D:4D—MSPSS	4.12/<0.001	0.263	[0.138, 0.388]
	BAI—SWLS	8.81/<0.001	0.512	[0.398, 0.626]
	BAI—MSPSS	8.15/<0.001	0.446	[0.339, 0.554]
	SWLS—MSPSSl	−0.81/0.418	−0.066	[−0.225, 0.093]
Female (*n* = 246)	NC—R-2D:4D	2.99/0.003	0.125	[0.043, 0.208]
	NC—BAI	−2.53/0.012	−0.052	[−0.092, −0.012]
	NC—SWLS	11.13/<0.001	0.574	[0.473, 0.675]
	NC—MSPSS	6.55/<0.001	0.368	[0.257, 0.478]
	R-2D:4D—BAI	−4.47/<0.001	−0.177	[−0.255, −0.100]
	R-2D:4D—SWLS	7.24/<0.001	0.448	[0.327, 0.570]
	R-2D:4D—MSPSS	3.42/0.001	0.242	[0.103, 0.381]
	BAI—SWLS	12.56/<0.001	0.626	[0.528, 0.723]
	BAI—MSPSS	7.32/<0.001	0.419	[0.307, 0.532]
	SWLS—MSPSS	−2.54/0.011	−0.206	[−0.365, −0.047]

Note: NC = neck circumference; R-2D:4D = right hand 2D:4D digit ratio; BAI = body adiposity index; SWLS = Satisfaction with Life Scale; MSPSS = Multidimensional Scale of Perceived Social Support.

## Data Availability

The data presented in this study are available upon request from the corresponding authors. The data are not publicly available due to privacy restrictions and ethical restrictions.

## Abbreviations

BMI	Body Mass Index
BAI	Body Adiposity Index
2D	2nd digit (index finger)
4D	4th digit (ring finger)
R-2D:4D	Right Hand 2D:4D Ratio
NC	Neck Circumference
W	Weight
H	Height
HC	Hip Circumference
ANOVA	Analysis of Variance
ROC	Receiver Operating Characteristic
AUC	Area Under the Curve
CI	Confidence Interval
SD	Standard Deviation
SWLS	Satisfaction with Life Scale
MSPSS	Multidimensional Scale of Perceived Social Support
